# Simulated eutrophication and browning alters zooplankton nutritional quality and determines juvenile fish growth and survival

**DOI:** 10.1002/ece3.3832

**Published:** 2018-02-06

**Authors:** Sami Johan Taipale, Kimmo Kalevi Kahilainen, Gordon William Holtgrieve, Elina Talvikki Peltomaa

**Affiliations:** ^1^ Department of Biological and Environmental Science University of Jyväskylä Jyväskylä Finland; ^2^ Faculty of Biosciences, Fisheries and Economics The Norwegian College of Fishery Science UiT The Arctic University of Norway Tromsø Norway; ^3^ School of Aquatic and Fishery Sciences University of Washington Seattle WA USA; ^4^ Department of Environmental Sciences University of Helsinki Helsinki Finland; ^5^ Lammi Biological Station University of Helsinki Lammi Finland

**Keywords:** amino acids, compound‐specific stable isotopes, essential biomolecules, fatty acids, food web, nutritional quality

## Abstract

The first few months of life is the most vulnerable period for fish and their optimal hatching time with zooplankton prey is favored by natural selection. Traditionally, however, prey abundance (i.e., zooplankton density) has been considered important, whereas prey nutritional composition has been largely neglected in natural settings. High‐quality zooplankton, rich in both essential amino acids (EAAs) and fatty acids (FAs), are required as starting prey to initiate development and fast juvenile growth. Prey quality is dependent on environmental conditions, and, for example, eutrophication and browning are two major factors defining primary producer community structures that will directly determine the nutritional quality of the basal food sources (algae, bacteria, terrestrial matter) for zooplankton. We experimentally tested how eutrophication and browning affect the growth and survival of juvenile rainbow trout (*Oncorhynchus mykiss*) by changing the quality of basal resources. We fed the fish on herbivorous zooplankton (*Daphnia*) grown with foods of different nutritional quality (algae, bacteria, terrestrial matter), and used GC‐MS, stable isotope labeling as well as bulk and compound‐specific stable isotope analyses for detecting the effects of different diets on the nutritional status of fish. The content of EAAs and omega‐3 (ω‐3) polyunsaturated FAs (PUFAs) in basal foods and zooplankton decreased in both eutrophication and browning treatments. The decrease in ω‐3 PUFA and especially docosahexaenoic acid (DHA) was reflected to fish juveniles, but they were able to compensate for low availability of EAAs in their food. Therefore, the reduced growth and survival of the juvenile fish was linked to the low availability of DHA. Fish showed very low ability to convert alpha‐linolenic acid (ALA) to DHA. We conclude that eutrophication and browning decrease the availability of the originally phytoplankton‐derived DHA for zooplankton and juvenile fish, suggesting bottom‐up regulation of food web quality.

## 1 INTRODUCTION

Eutrophication and browning are major aspect of environmental change in freshwaters with potentially large impacts on ecosystem functioning (Monteith et al., 2007; Scheffer & Carpenter, 2003; Schindler, 2012). Eutrophication of freshwater ecosystems has been intensively studied since 1970s with well‐known shifts toward increased pelagic phytoplankton production, decreased photic layer depth with reduced benthic production (Schindler, 1974, 2012). Generally speaking, eutrophication drives phytoplankton communities from a predominance of diatoms toward cyanobacteria and green algae dominance (Taipale, Vuorio, et al. 2016). Ecosystem browning—the increase in dissolved organic carbon (DOC) and particulate organic matter (POM) of catchment origin—increases light attenuation and decreases the photic layer depth, generally shifting ecosystems away from autotrophic, phytoplankton production toward increasing heterotrophic bacterial production (Forsström, Roiha, & Rautio, 2013; Karlsson, Bergström, Byström, & Lee Hein, 2015; Karlsson et al., 2009). Both eutrophication and browning have been observed to reduce biodiversity across multiple trophic levels (Karlsson et al., 2009; Vonlanthen et al., 2012). While the effects of eutrophication and browning on basal production is generally well‐understood, how shifts in basal resource pathways impact the availability of essential biomolecules, and their transfer through the food web to higher consumers, has only recently received consideration (Galloway & Winder, 2015; Paulsen, Clemmesen, & Malzahn, 2014; Taipale, et al. 2016).

The nutritional requirements of fish to optimize growth and reproduction are well‐known from extensive studies in aquaculture (e.g., Food and Aquaculture Organization of the United Nations, hereafter FAO). Most fish require relatively high dietary protein content for optimal growth (Weatherley & Gill, 1983); this is especially true for fish larvae (Ronnestad, Thorsen, & Finn, 1998; Wilson & Halver, 1986). Fish, like all animals, cannot synthesize essential amino acids (EAAs) de novo (Ketola, 1982). The nutritional value of a food item to a consumer is therefore generally considered to be high if its composition of EAAs is close to the consumer's own tissues (Brown, Jeffrey, Volkman, & Dunstan, 1997).

Polyunsaturated fatty acids (FAs), especially omega‐3 (ω‐3) and omega‐6 (ω‐6) FAs are another well recognized group of essential biomolecules for animals (Arts, Brett, & Kainz, 2009). In mammals, birds, and fish, EPA (20:5ω3) and DHA (22:6ω3) are required for growth, eye and brain tissue development and immunity function, and are thus crucial biomolecules for juveniles (Tocher, 2010). However, EPA and DHA are synthesized only by few phytoplankton taxa, that is, dinoflagellates, golden algae, diatoms, and cryptomonads (Galloway & Winder, 2015; Taipale, et al. 2016), and consumers need to obtain them either directly from the diet or convert them from precursor FAs such as linoleic (LIN; 18:2ω6) and alpha‐linolenic acid (ALA, 18:3ω3; Arts et al., 2009). The conversion efficiency of EPA and DHA from ALA varies greatly among aquatic and terrestrial animals (Arts et al., 2009; Burdge & Calder, 2005; Taipale, Brett, & Kainz, 2011), and also between different developmental stages (Tocher, 2010). For example, adult rainbow trout (*Oncorhynchus mykiss*) are able to synthesize EPA and DHA from high concentration of dietary ALA (Gregory & James, 2014), but having a direct dietary source of DHA is crucial for larvae (Wirth, Sfeffens, Meinelt, & Steinberg, 1997). Due to differences in feeding preferences and metabolism, EPA is naturally abundant in cladocerans (e.g., *Daphnia*) and DHA in copepods (e.g., *Eudiaptomus*; Brett, Müller‐Navarra, & Persson, 2009; Hiltunen, Strandberg, Keinänen, Taipale, & Kankaala, 2014; Hiltunen, Taipale, Strandberg, Kahilainen, & Kankaala, 2016). Due to the high preferences of DHA of copepods and fish, DHA is highly enriched in many aquatic food webs (Strandberg et al., 2015).

Information on optimal fish growth is difficult to translate to consumer dynamics in natural ecosystems however, in part because relatively little is known about the quality of natural fish diets in response to basal production pathways and how key biomolecules in natural fish prey transfer to consumers across ecosystem types. Freshwater microalgae synthesize all nine EAAs that are required by higher trophic levels for protein synthesis (Ahlgren, Gustafsson, & Boberg, 1992; Peltomaa, Aalto, Vuorio, & Taipale, 2017). Protein content of microalgae is also high (>50% of dry weight). Both cyanobacteria and green algae are unable to synthesize essential FAs including EPA and DHA, and thereby lower the availability of these biomolecules in the food web (Galloway & Winder, 2015; Müller‐Navarra et al., 2004; Persson, Brett, Vrede, & Ravet, 2007; Taipale et al., 2013; Taipale, et al. 2016). Heterotrophic bacteria feeding on terrestrial organic matter lacks essential fatty acids and sterols, and feeding trials indicate a terrestrial‐based energy pathway is inadequate for growth of zooplankton (Brett, Kainz, Taipale, & Seshan, 2009; Martin‐Creuzburg, Beck, & Freese, 2011; McMeans, Koussoroplis, Arts, & Kainz, 2015; Taipale, et al. 2016). In natural ecosystems, increased heterotrophic production with browning is often associated with changes in the phytoplankton community composition toward increasing cryptophytes and raphidophytes, both of which are EPA and DHA synthetizing taxa (Ask, Karlsson, & Jansson, 2012; Taipale, et al. 2016; Weyhenmeyer, Willén, & Sonesten, 2004). Despite general awareness of differences in the availability of essential AAs and FAs across production pathways, the transfer of these key biomolecules from producers to primary consumers (zooplankton) to juvenile fish has not been yet studied.

Juvenile fish frequently feed on multiple zooplankton taxa including rotifers, cladocerans (*Daphnia*), and copepods (Perga, Bec, & Anneville, 2009; Turner, 2004). These taxa are nutritionally different to consumers due to their own feeding preferences and the quality of consumed basal food (phytoplankton, bacteria, and/or terrestrial POM), and both abundance and reproduction of crustacean zooplankton in natural lakes can limit the quality of their diet. A high‐quality diet for crustacean zooplankton simultaneously includes high amounts of EAAs and PUFAs (Peltomaa et al., 2017). Intermediate quality diet for zooplankton includes all essential biomolecules, but in low amounts, whereas poor quality diet lacks some of the essential biomolecules. *Daphnia* can achieve high somatic growth with high or intermediate quality diet, whereas high reproduction rate requires high amounts of all essential biomolecules (Peltomaa et al., 2017). Generally, cryptophytes, synurophytes, and diatoms are high‐quality food for *Daphnia*, whereas green algae are intermediate quality due to the lack of EPA (Peltomaa et al., 2017). Cyanobacteria, bacteria, and tPOM are poor quality food, but when mixed with phytoplankton, they can support the somatic growth of *Daphnia* (Martin‐Creuzburg et al., 2011; McMeans et al., 2015; Taipale, Brett, Pulkkinen, & Kainz, 2012; Taipale et al., 2014; Wenzel et al., 2012). However, bacteria can contain high amounts of proteins and thus may support zooplankton growth better than direct consumption of tPOM (Taipale et al., 2014).

The ^13^C labeling experiments (^13^CLE) has been established as an effective method for determining food web structure and transfer of individual molecules from diet to consumer (Cole, Carpenter, Kitchell, & Pace, 2002; Cole et al., 2006; Taipale, Kankaala, Hämäläinen, & Jones, 2009; Taipale et al., 2011). Phytoplankton is usually labeled with an unnatural abundance of ^13^C relative to ^12^C, and the presence of this enriched ^13^C is tracked through multiple components of the food web. This isotopic labeling allows for better estimates of proportion diet composition in consumers using isotope mixing models (e.g., IsoSource, SIAR or SIBER; Phillips & Gregg, 2001; Parnell, Inger, Bearhop, & Jackson, 2010; Jackson, Inger, Parnell, & Bearshop, 2011). A previous ^13^CLE study showed *Daphnia* fatty acid turn‐over rate to be about 6 days and that *Daphnia* are unable to synthesize EPA from ALA efficiently (Taipale et al., 2011). To date, ^13^CLE has not been used for studying FA turnover rates in fish or ability to bioconversion of DHA from ALA.

In this study, we performed feeding experiments under simulated eutrophication and browning conditions to quantify the effect of sifting basal resource pathways on the quality of zooplankton as prey to their predators (juvenile rainbow trout. Diet quality was assessed by quantifying the transfer of essential biomolecules using combined approaches of essential AA and FA abundance with ^13^CLE followed by bulk and compound‐specific isotope analysis of essential FA analyses. We hypothesized that increasingly severe browning will reduce transfer of essential AAs more than eutrophication (H1). Secondly, we hypothesized that both eutrophication and browning will reduce the accumulation of ω‐3 PUFAs in zooplankton, but 4‐week‐old trout juveniles are able to compensate for this reduction by converting EPA and DHA from ALA (H2). Thirdly, we anticipated that joint reduction of EAAs and FAs at the basal trophic level has cascading negative effects causing decreased growth and survival of fish juveniles (H3).

## 2 METHODS

### 2.1 Preparation of basal resources: phytoplankton, bacteria, and tPOM

We selected a fast‐growing green‐algae *Acutodesmus* sp. (isolated from Lake Basel) that represents an intermediate nutritional quality algae in lakes; green algae typically contain high amounts of EAA and ALA, but lack EPA and DHA (Peltomaa et al., 2017). *Acutodesmus* was cultured in modified Woods Hole CHU 10 medium (MWC, Guillard, 1975) and ^13^C‐labelled with 5% of NaH^13^CO_3_ (99%, Cambridge Isotope Laboratories, Cambridge, UK) to achieve a target ^13^C‐enrichment of 50‰ vs. VPDB. Additionally, NaH^13^CO_3_ was added once a week to the *Acutodesmus* culture to maintain ^13^C‐label at a roughly consistent level. The high‐quality diet in the experiments was represented by *Cryptomonas ovata* (SPCC K‐1876), which is rich in EAA, ALA, and EPA. *C. ovata* was cultured in Artificial Freshwater medium (AF6, Watanabe, Kawachi, Hiroki, & Kasai, 2000). Both phytoplankton strains were maintained at 18–20°C under a 14:10 hours or 16:8 hours of light:dark cycle with light intensity of 30–70 μmol m^−2^ s^−1^. The heterotrophic gram‐positive bacterium, *Micrococcus luteus* (ATCC 4698)*,* was selected to represent typical heterotrophic freshwater bacteria in boreal lakes. *M. luteus* was cultivated using tryptic soy broth media in serum vials (150 ml) at 30°C for 48–60 hr, with new cultures started from plate colonies every second day. Fallen birch leaves (*Betula pendula*) were used as a terrestrial matter (tPOM) source for boreal lakes. Birch leaves were ground to fine particles using a Retch ZM 100 GWB ultra centrifugal mill and diluted then to modified Woods Hole (WC) medium and filtered through a 50 μm screen.

### 2.2 Zooplankton culturing

For all treatments, we used *Daphnia magna* clone DK‐35‐9 (hereafter *Daphnia*) initially raised and maintained on *Acutodesmus*. *Daphnia* were cultured in 1 L beakers using ADAM medium and fed every second day from the basal resource cultures. Due to the high required biomass of *Daphnia* for each fish feeding experiment, we were not able to culture the required *Daphnia* across all treatments at the same time, but rather one treatment at a time. Total culturing time for *Daphnia* in each treatment was 2 months. Two size‐classes of *Daphnia* were produced for the experiments: neonate *Daphnia* (juveniles of adult *Daphnia* fed on each basal resource culture >12 days) were harvested after 4–6 days of culturing (size <0.5 mm) and adult (size = 1–2 mm) *Daphnia* were harvested after 12–16 days of culturing. *Daphnia* were stored at −80°C until given to juvenile trout.

### 2.3 Juvenile fish

Rainbow trout juveniles were obtained from a fish hatchery at 1 month old where they were fed standard fish feed also used in our experiments as a control. Before that the fish juveniles had been using their yolk sacs and had not been fed. Rainbow trout juveniles were placed individually in 0.5‐L liter plastic tanks with continuous flow of oxygenized water (temperature 15 ± 1°C; light cycle 10 L:14 D). Each treatment had four replicates. The average (±*SD*) wet weight of all juveniles at the beginning was 96 ± 19 mg. Tanks were flushed with filtered and aerated tap water at rate 0.1 L/min. Aeration was stopped during fish feeding in the morning (8 a.m.) and evening (5 p.m.) for 15 min. Food was provided in excess, and we controlled that all juvenile individuals fed on the provided food in all treatments. During the first 14 days, juvenile trout were fed on juvenile *Daphnia* (size <0.5 mm) which after rainbow trout larvae were fed with adult *Daphnia* (size = 1–2 mm). Rainbow trout has relatively large gape size and thus able to feed on provided prey sizes. This was further verified daily by visual observations of prey consumption. The feeding experiments lasted for 21 days.

### 2.4 Experimental design of the three trophic level experiment

**Figure 1:**
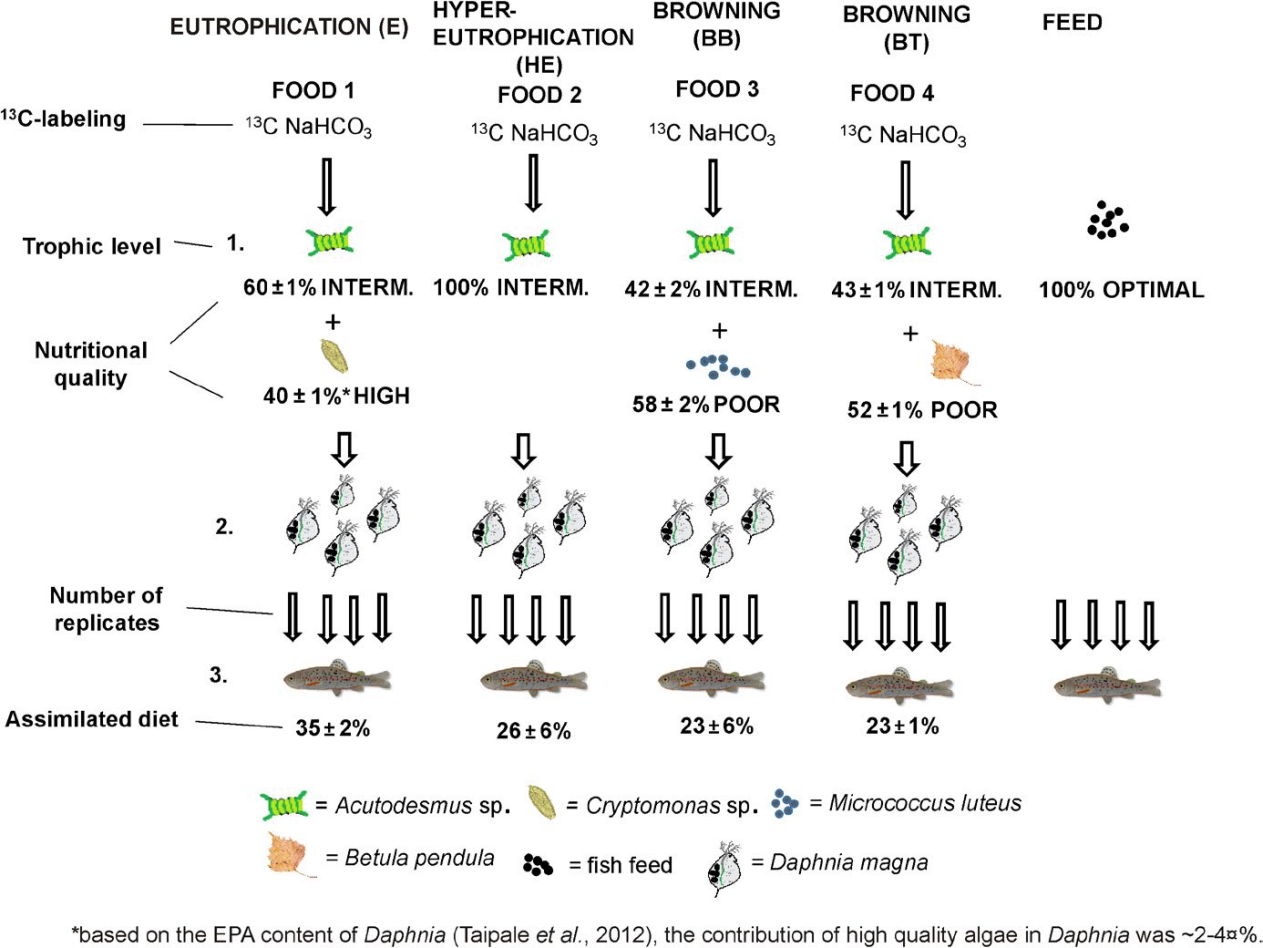
Diagram of the experimental design, which included three trophic levels: basal resource (algae, bacteria, or tPOM), herbivorous zooplankton (*Daphnia magna*), and juvenile trout (*Oncorhynchus mykiss*). Treatments aimed to simulate the effects of eutrophication and browning on the nutritional quality of the lake food web, with treatments E and HE simulating eutrophication and BB and BT simulating browning. This was achieved by feeding herbivorous zooplankton from different basal resource cultures in varying proportions. Treatment E was the mix of algae of high (*Cryptomonas ovata)* and intermediate (*Acutodesmus* sp.) diet quality. HE contained solely intermediate quality algae (*Acutodesmus*). BB (browning with bacteria) was the mix of intermediate quality algae (*Acutodesmus*) and poor quality bacteria (*Micrococcus luteus*). BT (browning with terrestrial input) was the mix of intermediate quality algae and poor quality tPOM (the ground leaves of birch: *Betula pendula*). *Daphnia* raised on these diets we then fed to rainbow trout and compared to trout raised on commercial fish feed (optimal diet). Each treatment included four replicates. The ^13^C‐isotope‐labeling of *Acutodesmus* was used for determining transfer of individual molecules from diet to consumer. The contribution of any two diets (mean ± *SD*) in *Daphnia* was estimated using two source mixing model calculation based on ^13^C/^12^C (Table 4)

*Daphnia* were grown under four different feeding treatments representing different lake types within hypotheses H1 and H3 (Figure 1). The feeding treatments were as follows: (1) eutrophic lakes (E) consisting of a mix of *Acutodesmus* and *C. ovata* (high in ALA, EPA and EAA); (2) hypereutrophic lakes (HE) with *Acutodesmus* only (high in ALA and EAA); (3) browning humic lake with bacteria (BB) was a mix of *Acutodesmus* and *M. luteus* (low in ALA and moderate in EAA); and browning humic lake with terrestrial input (BT) with a mix of *Acutodesmus* and tPOM (low in ALA and EAA). Additionally, a set of rainbow trout juveniles were fed standard fish feed (Vita Fry Feed with pellet size of 0.5 mm, Raisioagro, Finland), which is developed for maximizing the growth and survival of juvenile fish for aquaculture production with unnaturally high concentrations of AAs, FAs, and other biomolecules (Tocher, 2010).

### 2.5 Biochemical and isotopic analyses of basal resources and consumers

We determined the proportional composition of AAs and FAs of the basal food, *Daphnia*, and rainbow trout juveniles. We further used ^13^C‐enriched sodium bicarbonate (NaHCO_3_) to isotopically distinguish *Acutodesmus* from the other basal resources and track the transfer of carbon fixed by each resource to *Daphnia* and juvenile fish. This procedure allowed the proportion of the supplied diet assimilated by the consumer to be estimated with greater accuracy than normal by widening the isotope difference between the two diets. To determine whether juvenile rainbow trout was able to convert DHA from ALA, fish were fed an ^13^CLE enriched ALA diet and compound‐specific stable isotope analysis of individual fatty acids used to detect if the ^13^C label was transferred to DHA within the fish.

#### 2.5.1 Bulk stable isotope analysis

We analyzed bulk tissues from all trophic levels across the experiment for ^13^C/^12^C (Figure 1). Following Taipale et al. (2014), ~0.6–1.2 mg of freeze‐dried phytoplankton, bacteria, tPOM, zooplankton, or fish sample was weighted and encapsulated to tin cups. The ^13^C/^12^C was measured using an Carlo‐Erba Flash 1112 series elemental analyze connected to a Thermo Finnigan Delta Plus Advantage isotope ratio mass spectrometer in continuous flow mode. Isotopic data are presented in standard delta notation with units of per mil (‰) and relative to the Vienna Pee Dee Belemnite (VPDB) international standard. Precession and accuracy were determined through repeated measurements of an internal working standard and were found to be 0.2 and 0.3, respectively.

#### 2.5.2 Biochemical, FA, and EAA analysis

Carbohydrate, lipid, and protein content in each trophic level were analyzed using described in Taipale, et al. 2016. Briefly, total carbohydrate content was analyzed using Dubois, Gilles, Hamilton, Rebers, and Smith (1956) protocol. Total protein content was analyzed by multiplying elemental nitrogen content with known nitrogen content of proteins. Here, we used 6.8 for phytoplankton, bacteria and tPOM (Lourenco, Barbarion, Lavin, Lanfer, & Aidar, 2004), 6.3 for zooplankton, and 5.6 for fish (Postel, Fock, & Hagen, 2000). Total lipids were measured gravimetrically by extraction of 1–5 mg freeze‐dried sample with chloroform:methanol:water (2:1:0.75). Fatty acids were isolated by transmethylation with 1% sulfuric acid in methanol. Resulting fatty acid methyl esters were identified and quantified by coupled gas chromatography‐mass spectrometery (GC‐MS, Shimadzu Ultra, Kyoto, Japan) using an Agilent^®^ DB‐23 column (30 m × 0.25 mm × 0.25 μm, Taipale, Hiltunen, Vuorio, & Peltomaa, 2016).

Individual amino acids were quantified by first hydrolyzing 0.5–1 mg of tissue with 1 ml of 6 mol/L HCl at 110°C for 20 hr. After the hydrolysis, the samples were diluted with 5 mL of deionized water and purified with Bio‐Rad Poly‐Prep Prefilled Chromatography Columns (cat # 731‐6213). Salts and organic compounds were removed by eluting with 10 ml deionized water (ion‐free), after which amino acids were eluted from the column with 6 ml of 2 mol/L of NH_4_OH. Recovered samples were dried under a gentle stream on N_2_ flow on heat block at 60°C. Amino acids were next derivatized to propyl cloroformates using commercial EZ:faast kits for preparation (Phenomenex). Dervatized samples were analyzed by GC‐MS using ZB‐AAA column (9.5 m × 0.25 μm × 0.25 mm) and the following temperature program: rise from initial temperature of 110°C to 320°C at rate 30°C/min, after which hold for 7 min at 320°C. Injection temperature was 300°C and interface 290°C. Total column flow was 2.35 ml/min and linear velocity 71.2 cm/s. Amino acid identification was based on specific ions included in the EZ:faast library. For quantification we used Sigma‐Aldrich AA‐18 standard mix of which we made four‐point calibration curve (0.005, 0.05, 0.1, 0.2 μg/μl) also using the same derivatization methods. Due to the properties of the EZ:faast kit, we were able to analyze eight EAAs (histidine, isoleucine, leucine, methionine, phenylalanine, threonine, valine, and lysine), but not tryptophan. We were also able quantify nine non‐EAAs (alanine, asparagine, glutamic acid, glycine, glycine‐proline, ornithine, proline, serine, and tyrosine), but not arginine.

#### 2.5.3 Compound‐specific stable isotope analysis

Direct measurement of ^13^C/^12^C from individual fatty acids allows the possibility of tracking the transfer of these metabolically important compounds through the food web, and combined with ^13^CLE, overall accuracy and specificity improves. The ^13^C/^12^C of dominant FAs present in trout muscle tissue was determined using a gas chromatography–combusiton–isotope ratio mass spectrometry. Analyses were performed at the University of Washington on Thermo‐Finnigan Trace Ultra GC coupled to a Delta V plus IRMS via a GC‐Isolink combustion interface. Fatty acids were separated using a 60‐m DB‐23 column (0.25 mm × 0.15 mm) and then oxidized to carbon dioxide in an oxidation reactor at a temperature of 940°C with the reduction reactor kept at 630°C. The injector temperature was kept at 270°C. The temperature program of the GC column started at 50°C and was kept for 1 min at 50°C, after which the temperature was raised by 30°C/min to 140°C, then by 1°C/min to 220°C, and finally by 15°C/min to 300°C. The total run time was 94.3 min. Only peaks with a total height of 50 mV at mass 44 were used in the analysis. The samples were run against an internal standard mix of six certified fatty acid standards (purchased from Sigma‐Aldrich) with known δ^13^C from independent elemental analysis. Of the six FAs, methyl tridecanoate (δ^13^C = −30.56‰), pentadecanoate (δ13C=‐29.274‰), and nonadecanoate (δ^13^C = −29.854‰) were used for standardization and drift correction. The calculated precision for standard FAME was ±0.4‰, and the accuracy was ±0.3‰. The δ^13^C value of each individual FA was corrected to account for a single carbon atom from methanol (−53.2 ‰) that is added during transmethylation using the following formula:

Final δ^13^C of value of FA = ((number of C in FAME + δ^13^C value of FAME) − (δ^13^C of methanol))/number of C in FA.

### 2.6 Fish growth rate

Specific growth rate (SGR) was used to calculate individual fish growth during the experiment:

$$\text{SGR}=\left(\left(\ln\left(W_{2}\right)-\ln\left(W_{1}\right)\right)\times 100\right)/t_{2}-t_{1},$$

where *W*_1_ and *W*_2_ are the weights (mg wet weight) at the beginning and end of experiment, *t*_1_ − *t*_2_ denotes the duration of experiment in days (21). Units are % body wet weight gain per day.

#### 2.6.1 Stable isotope mixing model

We calculated the proportional contribution of each basal resource to consumer tissues using a two‐source carbon isotope mixing model (IsoError software, version 1.04; Phillips & Gregg, 2001). The mean proportion of source A in a mixture (*f*_*A*_) = δ_M_ − δ_B_/δ_A_ − δ_B_, where δ_*M*_, δ_*A*_, and δ_*B*_ represent the mean isotopic signatures (e.g., δ^13^C) for the mixture *M* and sources *A* and *B* (Phillips & Gregg, 2001). Error estimates are included and based on measured variance in source and mixture populations; replicate measurements (*n* = 3) for diets and *Daphnia* in different treatments were used in the calculations.

We used δ^13^C measurements and two source mixing modeling also to see, if nutritional quality of given food has influence on carbon turnover rate in juvenile rainbow trout. For these calculations, A was the contribution of ingested *Daphnia* by trout juveniles, B was old diet (fish feed) δ_M_ was δ^13^C value of trout juveniles, δ_A_ was δ^13^C value of *Daphnia*, and δ_B_ was δ^13^C value of muscle of trout juveniles before experiment. In all cases, we had only two diet sources, and thus, the uncertainty caused by variability of both sources was taken into account. For old carbon signal, we used the average of δ^13^C value and standard deviations of three replicate measurements of trout grown with fish feed.

In our third application, we a used two source mixing modeling to define how much of DHA originated from dietary ALA and how much of it represented DHA ingested before the experiment. In this case, A was the contribution of ALA from total DHA content of trout, δ_M_ was δ^13^C value of DHA in trout, δ_A_ was δ^13^C value of ALA in trout, and δ_B_ was δ^13^C value of DHA in trout muscle before experiment. For carbon signal of A and B, we used the average of δ^13^C value and standard deviations of two replicate measurements.

### 2.7 Statistical analysis

We explored hypothesis one (H1) by comparing the content of EAA and NEAA of zooplankton and rainbow trout juveniles among treatments with an one‐way ANOVA and Tukey's HSD test for pairwise comparisons. For testing H2 and transfer of ω‐3 PUFA in the food web, we compared ALA + SDA, EPA, DHA, the sum of HUFA ω‐3, total ω‐3 FA of zooplankton and trout among treatments. For testing eutrophication and browning impact on the transfer of ω‐6 PUFA, we compared total ω‐6 PUFA, LIN, and ARA content of second (*Daphnia* and feed) and third trophic level (juvenile trout) among different treatments. In case of equal variances, we employed one‐way ANOVA and Tukey's HSD test for pairwise comparisons; samples with unequal variances were tested with Welch ANOVA and Dunnet's T3 test. Finally, for the H3, we used Life Table method and Gehan's generalized Willcoxon test (Gehan, 1965) for comparing survival distributions of trout between treatments. Due to unequal variances, differences in the somatic growth of trout were tested using Welch ANOVA and Dunnet's T3 test. Limit of statistical significance in all tests was set to α ≤ 0.05. Statistical analyses were conducted using IBM SPSS (version 24.0; IBM 2016) software.

## 3 RESULTS

### 3.1 Structural content of diet and transfer of EAA and FA (H1)

#### 3.1.1 Protein, lipid, and carbohydrate content of basal resources, zooplankton, and trout

**Figure 2:**
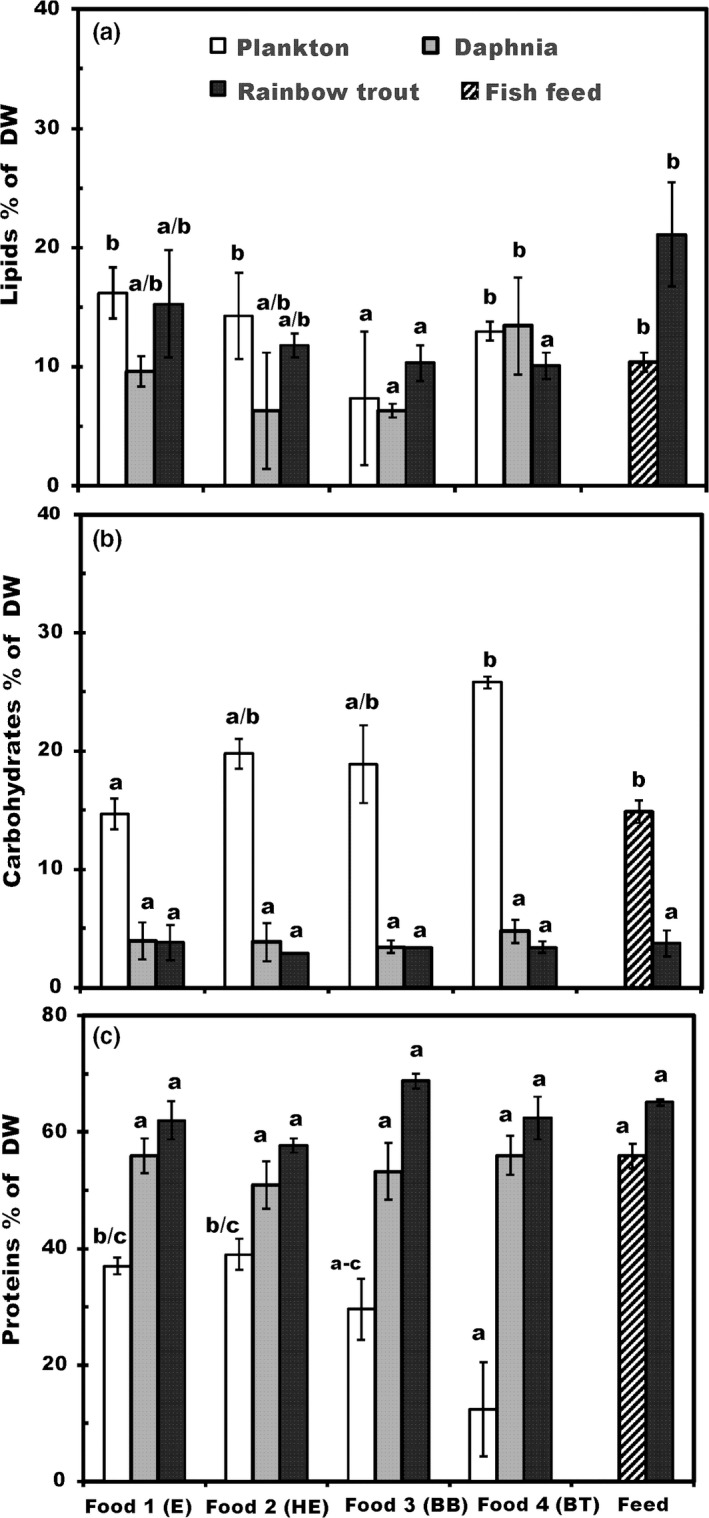
The (A) lipid, (B) carbohydrate, and (C) protein content (% of dry weight, mean ± 1 *SD*) of basal foods (1–4), *Daphnia* fed on basal foods, fish feed, and rainbow trout juveniles. The number of analyzed samples (*n*) is four for all groups. Different letters (a–c) denote significant differences (*p* < .05) between diets

Comparing within trophic level, the average lipid content among basal resources across the experimental treatments was 12.7 ± 4.1% (mean ± 1 *SD* of DW) with food 3 (BB) significantly lower than the rest (ANOVA <0.05; Table 1, Figure 2A). There was a significant difference in lipids in *Daphnia* between foods 3 and 4 (BB < BT), with no significant difference among the remaining foods including fish feed (Table 1). Finally, there was a significant difference in lipid content of juvenile fishes between fish feed and the two browning treatments (BB and BT), but no difference among the remaining foods. Comparing across trophic level, the lipid content was higher in feed than in *Daphnia* for food 3 (BB). Lipid content of the juvenile fish was significantly higher in fish feed treatments than in other treatments (ANOVA; *p* < .005; Table 1). The lipid content of *Daphnia* and fish feed was lower (9.2 ± 3.8%) than in basal foods, whereas the lipid content was highest (14.1 ± 5.0%) in trout.

**Table 1:** Statistical results from the comparison of means within trophic levels (basal, *Daphnia*/feed, trout) of different biomolecules (content; μg biomolecule/g DW)

Biomolecule	Organism	Analysis	*F*	*df*_1_	*df*_2_	*p*
Carbohydrate	Basal	Welch ANOVA	6.5	4	3.7	.02
	*Daphnia*	ANOVA	65.6	4	15	<.0001
	Trout	ANOVA	1.5	4	12	.258
Lipids	Basal	ANOVA	7.1	3	8	.012
	*Daphnia*	Welch ANOVA	17.1	4	6.7	.001
	Trout	Welch ANOVA	58.4	4	6.1	.001
Proteins	Basal	Welch ANOVA	22.7	3	6.2	.01
	*Daphnia*	ANOVA	1.6	4	15	.231
	Trout	Welch ANOVA	2.4	4	6.3	.155
ALA + SDA	Basal	ANOVA	21.1	3	15	<.0001
	*Daphnia*	ANOVA	65.9	4	10	<.0001
	Trout	ANOVA	31.5	4	5.7	<.001
EPA	Basal	Welch ANOVA	15.1	3	7.2	.002
	*Daphnia*	Welch ANOVA	18.1	4	4.0	.001
	Trout	ANOVA	52.6	4	13	<.0001
DHA	Basal	Welch ANOVA	43.5	3	7.1	<.0001
	*Daphnia*	Welch ANOVA	44.4	4	4.6	<.0001
	Trout	ANOVA	9.1	4	5.3	.01
LIN	Basal	ANOVA	9.2	3	15	.001
	*Daphnia*	Welch ANOVA	49.8	4	10	<.0001
	Trout	Welch ANOVA	20.6	4	5.2	.002
ARA	Basal					
	*Daphnia*	Welch ANOVA	214.9	4	10	<.0001
	Trout	ANOVA	16.0	4	5.8	.003
EAA	Basal	ANOVA	22.5	3	12	<.0001
	*Daphnia*	ANOVA	14.0	4	15	<.0001
NEAA	Basal	ANOVA	4.8	3	12	.020
	*Daphnia*	ANOVA	4.2	4	15	.017

Test was standard one‐way analysis of variance except in the case of unequal variances, in which case a Welch ANOVA was employed. Basal food sources did not contain arachidonic acid (ARA). Additionally, juvenile trout was not tested for amino acid content due to lack of sufficient sample. Other abbreviations are α‐linoleic acid (ALA), stearidonic acid (SDA), eicosapentaenoic acid (EPA), docosahexaenoic acid (DHA), linoleic acid (LIN), essential amino acids (EAA), and nonessential amino acids (NEAA).

The average carbohydrate content of basal foods was 19.8 ± 2.2%, which is fivefold higher than the carbohydrate content of *Daphnia* (4.0 ± 0.6%) and juvenile fish (3.6 ± 0.9%). Within trophic levels, the carbohydrate content was lowest in food 1 (E) and highest in food 4 (BT; Table 1, Figure 2B) for the basal resources. At the second trophic level, the carbohydrate content of fish feed was over three times higher *Daphnia* (Table 1), but there was no significant difference among basal resource treatments (Table 1). Lastly, the carbohydrate content of juveniles fishes did not differ significantly among the treatments (Table 1).

Within trophic levels, protein content varied greatest in the basal foods (Table 1, Figure 2C) due to the extremely low protein content (12.4 ± 8.1%) of food 4 (BT). The average protein content of the other remaining basal resources was 32.6 ± 5.5%. However, despite this difference, the protein content of *Daphnia* from all treatments as well as protein content of fish feed was statistically equivalent and exceeded the protein requirements (>45% of DW) for juvenile rainbow trout (Table 2). Similarly, the protein content of juvenile fish was high in all treatments (Figure 2C) and did not differ between the treatments.

**Table 2:** Dietary nutrient requirement of rainbow trout (*Oncorhynchus mykiss*) juveniles based on recommendation of Food and Agriculture Organization of the United Nations (FAO 2016) in comparison with biochemical content of diets from this experiment (% of DW)

	Requirement for juvenile	Food 1 (E)	Food 2 (HE).	Food 3 (BB)	Food 4 (BT)	Fish feed
Crude protein, % min of DW	45	51 ± 3.7	48 ± 3.5	51 ± 4.1	49 ± 3.6	54 ± 1.0
Essential amino acids, % min of DW						
Arginine	2	na	na	na	na	na
Histidine	0.7	0.04 ± 0.02	0.04 ± 0.04	0.04 ± 0.01	0.05 ± 0.01	0.07 ± 0.03
Isoleucine	0.8	1.0 ± 0.1	1.0 ± 0.03	0.7 ± 0.12	0.8 ± 0.1	0.7 ± 0.09
Leucine	1.4	2.4 ± 0.23	2.0 ± 0.19	2.1 ± 0.47	2.0 ± 0.13	1.4 ± 0.13
Lysine	1.8	2.0 ± 0.5	1.5 ± 0.14	1.1 ± 0.38	1.4 ± 0.30	1.5 ± 0.46
Methionine	1	0.7 ± 0.3	0.03 ± 0.004	0.6 ± 0.1	0.5 ± 0.05	0.5 ± 0.02
Phenylalanine	1.2	1.2 ± 0.1	1.1 ± 0.03	1.2 ± 0.2	1.0 ± 0.09	0.5 ± 0.02
Threonine	0.8	0.8 ± 0.03	0.8 ± 0.12	0.5 ± 0.19	0.8 ± 0.09	0.6 ± 0.03
Tryptophan	0.2	na	na	na	na	na
Valine	1.3	1.4 ± 0.08	1.3 ± 0.09	0.8 ± 0.13	1.0 ± 0.15	0.8 ± 0.18
Lipids, % of DW	na	10.3 ± 0.2	6.3 ± 0.5	6.3 ± 0.05	12.0 ± 0.3	10.2 ± 0.14
Essential fatty acids, % min of DW						
18:2ω‐6 (LIN)		0.8 ± 0.01	0.4 ± 0.09	0.6 ± 0.02	0.7 ± 0.06	0.4 ± 0.01
20:4ω‐6 (ARA)	0.5	0.1 ± 0.02	0.03 ± 0.01	0.11 ± 0.01	0.11 ± 0.01	0.09 ± 0.01
18:3ω‐3^a^ (ALA + SDA)	1^a^	2.5 ± 0.57	3.3 ± 1.1	1.4 ± 0.13	1.9 ± 0.13	0.7 ± 0.12
20:5ω‐3 (EPA)	1	0.15 ± 0.03	0.05 ± 0.01	0.05 ± 0.01	0.04 ± 0.01	1.6 ± 0.07
22:6ω‐3 (DHA)	0.5	0.05 ± 0.02	0.05 ± 0.01	0.06 ± 0.01	0.06 ± 0.01	1.8 ± 0.07
Carbohydrate, % max of DW	12	4.0 ± 1.6	3.8 ± 1.6	3.4 ± 0.5	4.8 ± 1.0	15 ± 1.0

Experiments with herbivorous zooplankton prey *Daphnia* were fed with Food 1 (E, eutrophication) was the mix of high (*Cryptomonas ovata*) and intermediate (*Acutodesmus* sp.) quality diets. Food 2 (HE, hypereutrophication) contained solely intermediate quality algae (*Acutodesmus* sp.). Food 3 (BB, browning with bacteria) was the mix of intermediate quality algae (*Acutodesmus* sp.) and poor quality bacteria (*Micrococcus luteus*). Food 4 (BT, browning with terrestrial input) was the mix of intermediate quality algae and poor quality tPOM (the ground leaves of birch: *Betula pendula*). Fish feed is commercial pellet (Vita 0.5 mm, Raisioagro, Finland) and treated as an optimal diet to fish juveniles. na, not analyzed. Essential fatty acid abbreviations: linoleic acid (LIN), arachidonic acid (ARA), alfa‐linolenic acid (ALA), stearidonic acid (SDA), eicosapentaaenoic acid (EPA), and docosahexaenoic acid (DHA).

a includes also SDA.

#### 3.1.2 Amino acids in three trophic levels

We were able to detect eight essential amino acids (EAA: histidine, isoleucine, leucine, lysine, methionine, phenylalanine, threonine, and valine) and nine nonessential amino acids (NEAA: alanine, asparagine, glutamic acid, glycine, glycine‐proline, ornithine, proline, serine, and tyrosine) from our samples (Table S1). We had low concentration of histidine for all samples, possibly as the result of poor recovery during analysis.

**Figure 3:**
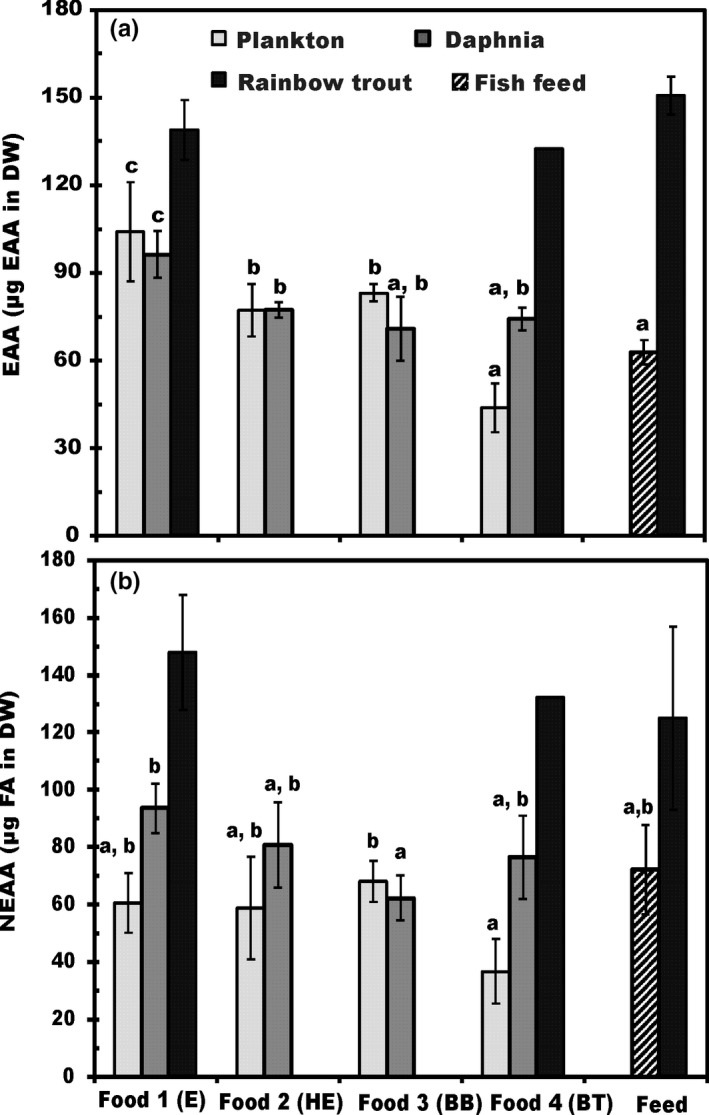
Percent abundance by mass (μg amino acids per mg DW ± 1 *SD*, see text for amino acid groups) of (A) essential amino acids (EAA) and (B) nonessential amino acids (NEAA) in three trophic levels (plankton, *Daphnia,* and juvenile trout). Number of analyzed samples (*n*) is four for basal foods, *Daphnia*, and fish fed fish feed. There were only two surviving replicates of fish under treatment HE (food 2) and only one for under treatment BT (food 4). There was insufficient sample for analysis of fish treatments HE and BB (foods 2 and 3). Different letters (a–c) denote significant differences (*p* < .05) between diets in amino acid content

The AA profile of basal food sources (foods 1–4) was similar. EAAs contributed 58 ± 4.2% of all AA. Leucine, lysine, proline, and phenylalanine were most abundant AA in basal food sources. The AA profile of *Daphnia* similarly did not differ among treatments. Leucine, alanine, lysine, and proline were most abundant AA in *Daphnia,* and the AA profile of *Daphnia* differed from fish feed only in glutamic acid (glutamic acid was 14.2 ± 3.2% of all AA in fish feed, but only 5.6 ± 2.3% in *Daphnia*). Glutamic acid is nonessential, and thus, EAAs as a proportion of all AAs was also higher in *Daphnia* (61 ± 4.2%) than in fish feed (47 ± 7.0%). The AA profiles of the juvenile trout were similar among treatments (with foods 1, 3 or fish feed) (Figure 3A) and EAA contributed 51 ± 2.1% of all AAs in fish. Alanine, leucine, glycine, and lysine were the four major amino acids in juvenile fish; however, fish receiving *Daphina* from treatment E (food 1) had less lysine than fish from treatment BT (food 4) or with commercial fish feed.

The EAA content of the basal foods differed greatly between the treatments (Figure 3A). The EAA content was highest in food 1 (E) and lowest in food 4 (BT) across basal foods. The same differences in the EAA contents were found on the second trophic level (Figure 3A). The content of EAA (% of dry weight) in *Daphnia* fed with foods 1–4 was close to or above the nutrient requirements of juvenile rainbow trout (Table 2). The EAA content juvenile fish was similar across treatments (1, 3 and feed).

Basal foods also differed in the content of NEAA. Food 4 has lowest NEAA content (37 ± 11.4 μg AA mg/C), whereas the content of NEAA was similar in foods 1‐3 and feed. In contrast to this, *Daphnia* with food 3 has lowest NEAA content, whereas *Daphnia* fed food 1 has highest. Nevertheless, the NEAA content of juvenile trout was similar across treatments.

### 3.2 Accumulation and bioconversion of FA (H2)

#### 3.2.1 ω‐3 polyunsaturated fatty acids across three trophic levels

Generally, ALA and SDA were the major ω‐3 PUFAs found in basal foods and in *Daphnia*, whereas DHA was the most abundant ω‐3 PUFA in fish feed and in trout juveniles. The dietary shift from fish feed to natural diets (zooplankton) influenced the FA‐profiles of the fish juveniles (Table S2); however, the contribution of EPA and DHA remained high in juveniles in all treatments. The contribution of EPA was highest in juveniles fed with fish feed than in juveniles fed on *Daphnia* diets (ANOVA: F_3,17_ = 37.6, *p* < .001). Despite this, there was no statistical difference in the DHA content of *Daphnia* among treatments (*p* = .247), although the proportion of DHA was higher in fish feed than *Daphnia*. The proportion of ALA was significantly larger in fish fed with foods 1 (E treatment) and 2 (HE treatment) than in other foods (ANOVA: *F*_3, 17_ = 43.8, *p* < .001; Table S2).

Among basal resources, the content of ALA and stearidonic acid (SDA) was highest in foods 1 (E) and 2 (HE) compared to foods 3 (BB) or 4 (BT). The same pattern was seen in the second trophic level with the content of ALA and SDA in *Daphnia* higher under food treatments 1 and 2 than compared to food treatments 3, 4, or the fish feed. However, ALA content exceeded the dietary requirements of rainbow trout juveniles in all treatments (>1% of DW, Table 1).

**Figure 4:**
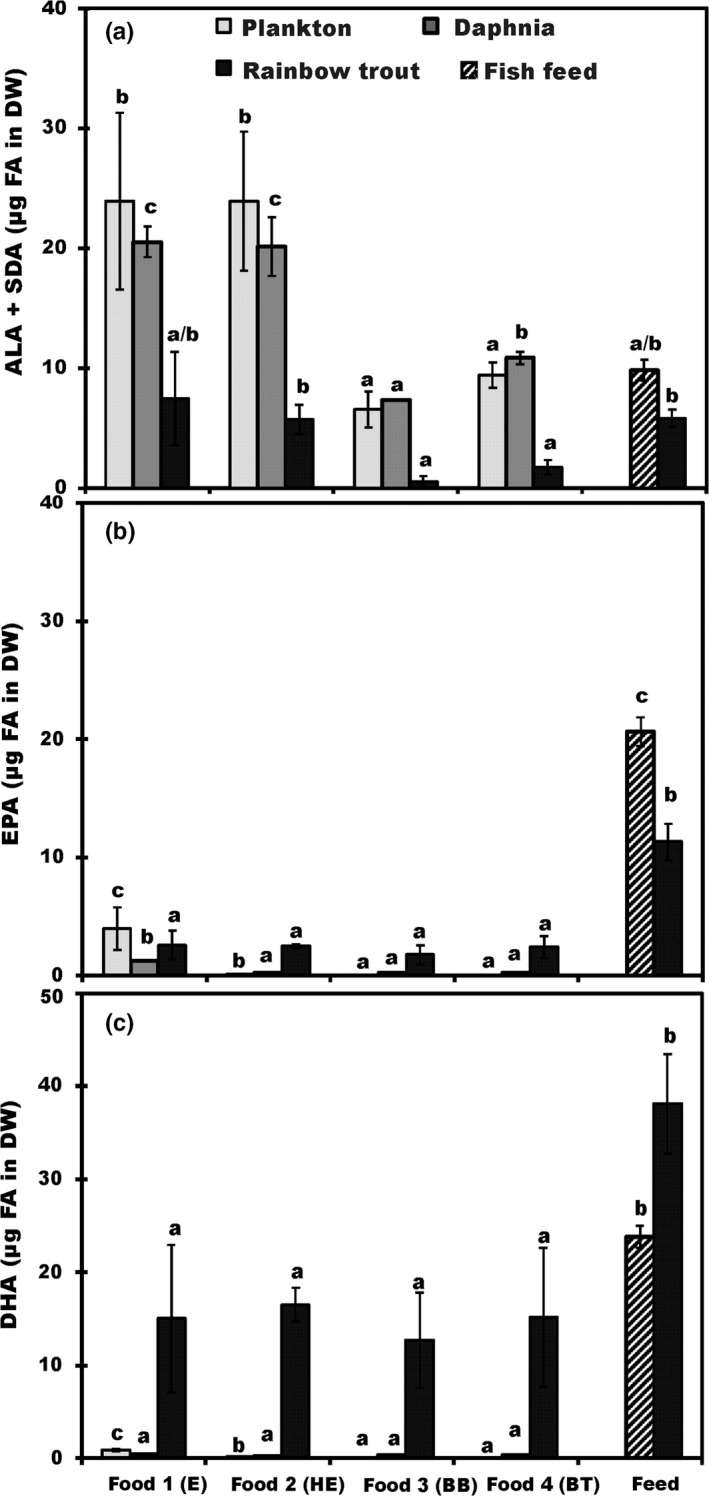
Percent abundance by mass (μg FA in mg DW ± *SD*) of (A) Alpha‐linolenic acid (ALA; 18:3ω3) and stearidonic acid (SDA; 18:4ω3), (B) eicosapentaenoic (EPA; 20:5ω3), and (c) docosahexaenoic (DHA; 22:6ω3) across three trophic levels (plankton, *Daphnia/*feed, and trouts). Number of analyzed sample (*n*) is four for all groups. Different letters (a–c) denote significant differences (*p* < 0.05) among diets

Among basal resources, the EPA content was highest in food 1 (E), whereas foods 2‐4 contained only trace amounts of EPA. However, the EPA content was statistically higher in food 2 (HE; *p* < .05) than in food 3 (BB) or in food 4 (BT; Figure 4B). For trophic level two, the EPA and DHA content of *Daphnia* was below dietary recommendation for juveniles rainbow trout and exceeded dietary recommendations only in fish feed. Total HUFA content (EPA + DHA) in trout juveniles decreased from pre‐experiment levels (23.8 ± 2.3 μg HUFA DW) for all *Daphnia* treatments (17.1 ± 6.2 μg HUFA DW), whereas their content doubled with the fish feed treatment (49.5 ± 6.9 μg HUFA DW).

#### 3.2.2 The ω‐6 polyunsaturated fatty acids in three trophic levels

**Figure 5:**
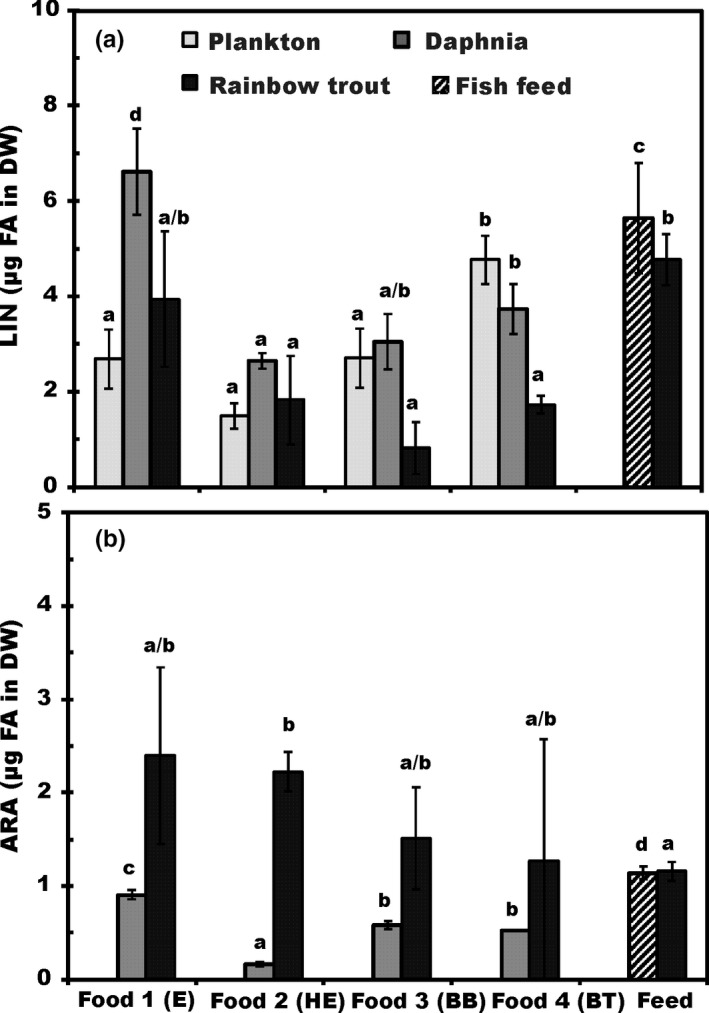
Percent abundance by mass (μg FA in mg DW ± *SD*) of (A) linoleic acid (LIN; 18:2ω6), and (B) arachidonic acid (ARA; 20:4ω6) across three trophic levels (plankton, *Daphnia/*feed, and trout juveniles). Number of analyzed sample (*n*) is 4 for all samples. Different letters (a–c) denote significant differences (*p* < .05) among diets in fatty acid content

LIN (18:2ω6) was only ω‐6 PUFA across the basal resources, whereas ARA (20:4 ω6) was found from second and third trophic level as well. LIN did not differ among the basal resource treatments (*p* > .11; Figure 5A). LIN was dominant ω‐6 FA in both *Daphnia* and fish feed, whereas the amount of ARA was below the recommendations for rainbow trout growth among all *Daphnia* treatments and the commercial feed (0.5% of DW, Table 2). LIN was also dominant ω‐6 FA in juvenile fish. LIN content of fish juveniles was highest in treatments of fish feed and food 1 (Figure 5A), whereas the ARA content of fish juveniles was similar in all treatments. The total content of ω‐6 FAs in juvenile fish was highest in the fish feed treatment and lowest in the poor quality diet (foods 3 and 4; Figure 5B), and thus, there was a significant difference in total ω‐6 FAs of trout juveniles across treatments (ANOVA: *F*_5, 12_ = 6.2, *p* = .004).

#### 3.2.3 The δ^13^C enrichment of bulk biomass and ω‐3 and ω‐6 PUFA in trouts

The green algae (*Acutodesmus* sp.) was labeled with 5% ^13^C bicarbonate for all experiments (Table 3), and bulk δ^13^C values of green algae varied from 25‰ to 300‰. The bulk biomass δ^13^C values of cryptophyte (*Cryptomonas ovata*), Actinobacteria (*Micrococcus luteus*) and grounded birch leaves (tPOM) differed greatly from ^13^C‐enriched values of green algae. The combined bulk biomass δ^13^C values of green algae and cryptophyte (food 1), bacteria (food 3), and tPOM (food 4) are shown in table 3, which varied from −21‰ to 135‰. In all treatments, *Daphnia* δ^13^C values were more enriched than natural δ^13^C values of cryptophyte, bacteria, or tPOM, thus showing assimilation of ^13^C‐enriched green algae into tissues. The δ^13^C values of *Daphnia* were close to the δ^13^C values of mix of basal with food 1. However, the δ^13^C values of *Daphnia* were more enriched than the δ^13^C values of mix of basal food sources with foods 3 and 4, showing preferential uptake of ^13^C‐labelled green algae than bacteria or tPOM. The δ^13^C value of trout was −21.6 ± 0.2‰ in control (fish feed) experiment. In relation to this control treatment, trout juveniles in all treatments were enriched with ^13^C thus showing trouts feeding on given food (Table 4). However, the ^13^C‐enrichment was higher with foods 1 (E) and 2 (HE) than with 3 (BB) and 4 (BT). According to two‐source mixing model (based on δ^13^C of bulk biomass), the 35 ± 2% and 26 ± 6% of organic carbon in trout originated from *Daphnia* in foods 1 and 2, respectively, whereas corresponding values for foods 3 and 4 were 23 ± 6% and 23 ± 1% (Table 4). Therefore, it can be concluded than the remaining of old isotope signal in the fish tissue was lowest (75 ± 2%) with food 1.

**Table 3:** Mean ± 1 *SD* ^13^C/^12^C (expressed as δ^13^C vs. VPDB) of bulk biomass from basal food sources (phytoplankton, bacteria, and tPOM), mixture of basal food sources (i.e., treatments), *Daphnia*, commercial fish feed, and juvenile rainbow trout

	The δ^13^C of	The δ^13^C of ω‐3 PUFA			The δ^13^C of ω‐6 PUFA	
	Bulk biomass	ALA	EPA	DHA	LIN	ARA
Food 1 (E)						
*Acutodesmus* sp.	258 ± 50					
*Cryptomonas ovata*	−27.9 ± 0.6					
Mix of basal	135 ± 10					
*Daphnia*	134 ± 10					
Trout	31.9 ± 6.8	86.9 ± 4.9	0.3 ± 7.3	−14.8 ± 3.7	134.2 ± 3.8	37.4 ± 4.3
Food 2 (HE)						
*Acutodesmus* sp.	25.4 ± 10					
*Daphnia*	39 ± 31					
Trout	−8.1 ± 2.9	14.0 ± 2.5	−8.4 ± 3.6	−17.5 ± 1.1	8.1 ± 10.5	5.5 ± 2.4
Food 3 (BB)						
*Acutodesmus* sp.	31 ± 5					
*Micrococcus luteus*	−21.2 ± 0.6					
Mix of basal	−17.8 ± 0.8					
*Daphnia*	0.5 ± 0.5					
Trout	−16.4 ± 0.8	−23.2 ± 5.8	−21.1 ± 6.0	−24.9 ± 3.3	−23.2 ± 5.8	−18.4 ± 4.1
Food 4 (BT)						
*Acutodesmus* sp.	121 ± 5					
tPOM	−29.5 ± 0.6					
Mix of basal	−21.3 ± 2.1					
*Daphnia*	35.5 ± 0.3					
Trout	−10.6 ± 0.4	14.7 ± 5.2	−20.2 ± 3.5	−25.3 ± 0.4	−0.2 ± 18.5	−16.3 ± 6.9
Feed						
Fish feed	−24.2 ± 0.3					
Trout	−21.6 ± 0.2	−32.5 ± 1.6	−28.0 ± 2.8	−25.8 ± 2.1	−36.5 ± 3.2	−23.2 ± 1.0

Also included is the δ^13^C of individual ω‐3 and ω‐6 PUFA from juvenile trout determined by GC‐C‐IRMS.

**Table 4:** IsoError were used for two source mixing model calculations where *A* and *B* indicate sources and *M* stands for mixture

	Treatment	*M*	*A*	*B*	δ_M_	δ_A_	δ_B_	f_A_	f_B_
Case 1	Food 1	*Daphnia*	*Acutodesmus* sp.	*Cryptomonas ovata*	134 ± 10	258 ± 50	−27.9 ± 0.6	57 ± 6	43 ± 6
	Food 3	*Daphnia*	*Acutodesmus* sp.	*Micrococcus luteus*	0.5 ± 0.5	31 ± 5	−21.2 ± 0.6	42 ± 2	58 ± 2
	Food 4	*Daphnia*	*Acutodesmus* sp.	tPOM	35.5 ± 0.3	121 ± 5	−29.5 ± 0.6	43 ± 1	57 ± 1
Case 2	Food 1	Trout	*Daphnia*	Old diet (fish feed)	31.9 ± 6.8	134 ± 10	−21.6 ± 0.2	34 ± 3	66 ± 3
	Food 2	Trout	*Daphnia*	Old diet (fish feed)	−8.1 ± 2.9	39 ± 31	−21.6 ± 0.2	22 ± 7	78 ± 7
	Food 3	Trout	*Daphnia*	Old diet (fish feed)	−16.4 ± 0.8	0.5 ± 0.5	−21.6 ± 0.2	24 ± 2	76 ± 2
	Food 4	Trout	*Daphnia*	Old diet (fish feed)	−10.6 ± 0.4	35.5 ± 0.3	−21.6 ± 0.2	19 ± 1	81 ± 1
Case 3	Food 1	DHA in trout	ALA in trout	DHA in feed trout	−14.8 ± 3.7	86.9 ± 4.9	−25.8 ± 2.1	10 ± 3	90 ± 3
	Food 2	DHA in trout	ALA in trout	DHA in feed trout	−17.5 ± 1.1	14 ± 2.5	−25.8 ± 2.1	21 ± 4	79 ± 4
	Food 3	DHA in trout	ALA in trout	DHA in feed trout	−24.9 ± 3.3	−23.2 ± 5.8	−25.8 ± 2.1	ns	ns
	Food 4	DHA in trout	ALA in trout	DHA in feed trout	−25.3 ± 0.4	14.7 ± 5.2	−25.8 ± 2.1	1 ± 4	99 ± 4

Correspondingly, δ_M_, δ_A_, and δ_B_ represent the mean isotopic signatures (δ^13^C) *M*,* A* and *B*, and *f*_*A*_ and *f*_*B*_ are the proportions of *A* and *B* in *M*. The δ^13^C of values are presented as mean ± 1 *SD* and proportions as mean ± 1 *SE*. Model result is from three different trials: case (1) proportion of ^13^C‐labelled *Acutodesmus* sp. (green algae) in *Daphnia*, case (2) proportion of *Daphnia* in trout muscle tissue and (3) the proportion of ALA in DHA in trout. See more details in Section 2. ns, no reasonable solution.

The δ^13^C value of ω‐3 and ω‐6 PUFA in juvenile trout given fish feed was more depleted than the bulk fish muscle (Table 3). In contrast to the fish feed diet, the δ^13^C value of ALA, LIN, and ARA in trout grown on foods 1, 2, and 4 was more ^13^C enriched than bulk fish muscle, while the δ^13^C value of ω‐3 and ω‐6 PUFA in trout from fish food 3 (BB) had very similar values to the fish feed trout and showed essentially no ^13^C enrichment relative to bulk tissues. The δ^13^C value of DHA of fish given fish feed was ^13^C enriched at −25.8 ± 2.1‰. In comparison, the DHA of trout grown under food treatments 3 and 4 was not enriched in^13^C and was only slightly in trout from food treatments 1 and 2 (Table 3). Based on the two‐source mixing model, only 10 ± 2% and 21 ± 3% of all DHA in juvenile fish from food treatments 1 and 2 originated from their zooplankton prey (Table 4), respectively.

### 3.3 Survival and somatic growth of trout (H3)

**Figure 6:**
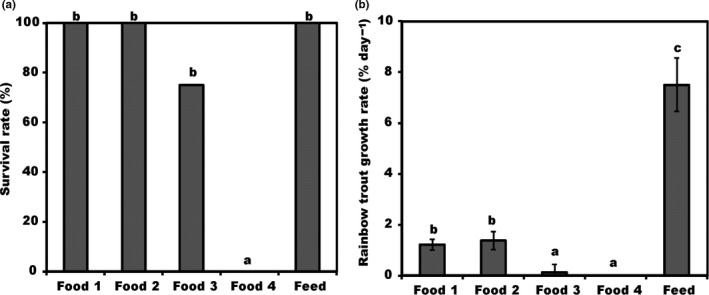
(A) Survival rate (%) and (B) specific growth rate of rainbow trout juveniles (% per day ±1 *SD*) given natural diets and commercial fish feed. Number of analyzed sample (*n*) is four for all groups. Different letters (a–c) denote significant differences (*p* < .05) between diets

Over the course of the 21‐day experiment all juvenile trout under food treatment 4 died (on days 4, 13, 15) and one fish juvenile died under food treatment 3 (BB) on day 19. As such, juvenile trout survival differed among treatments (Wilcoxon Gehan Statistic *F* = 10.9, *df *= 4, *p* = .028), but the pairwise comparisons showed that this was due to the zero survival rate for the BT treatment (Figure 6A).

The specific growth rate (SGR) of the juvenile rainbow trout also differed among the treatments (Welch ANOVA: *F*_3, 6.251_ = 755.278, *p* < .001, Figure 6B), where fish given commercial fish feed having significantly higher (Dunnett T3, *p* < .05) specific growth rate (7.0 ± 0.2% FW/day) compared to all other treatments. Additionally, trout under food treatments 1 and 2 had higher growth rate (1.4 ± 0.3 mg FW/day) than those in treatment 3 (0.14 ± 0.4 mg FW/day). As all individuals in treatment 4 (BT) died, the SGR could not be calculated.

## 4 DISCUSSION

### 4.1 The main findings

Our results showed that simulated eutrophication and browning had major influence on the nutritional quality of basal producers, with cascading impact through the freshwater food web. The content of essential amino acids and ω‐3 polyunsaturated fatty acid (PUFA) of basal foods and zooplankton decreased relative to nutritional quality of diets, with the decrease higher in browning than eutrophication treatments. The decrease of key lipids—ω‐3 PUFA and especially DHA—in basal food and zooplankton was reflected the lipid composition of fish juveniles, which were unable to desaturate and elongate ALA to DHA efficiently. In contrast, juvenile fish were able to compensate for low EAA availability in their diet by accumulation and retention. Prey nutritional quality influenced the survival and growth of juvenile fish directly, which were both lower in browning than in the eutrophication treatments. Taken collectively, our experimental evidence suggests that the decreasing nutritional value at the first trophic level under ecosystem change has intensifying negative effects on the upper trophic levels of lake food web.

### 4.2 Transfer of essential amino acids (H1)

High‐quality proteins and amino acids are especially important for the initial growth rate of juvenile fish, which is in turn linked to fish survival. While the protein content of the basal foods differed among the treatments, there was no difference at the next two trophic levels. Therefore, it seems that zooplankton and fish are able to obtain proteins from their diet very efficiently and indicates that our first hypothesis was only partially supported in the sense that browning with tPOM decreased the EAA content in the basal food more than eutrophication. However, browning with bacteria resulted in equal amount of EAAs than hyper‐eutrophication (food 3). *Daphnia* were able to balance the low availability of EAA in tPOM to some extent, which resulted in similar amount of EAAs in *Daphnia* under the browning and the hyper‐eutrophication scenarios. The small addition of high‐quality food (cryptophyte) under the eutrophication scenario substantially increased the EAA content in the basal foods and in *Daphnia*, demonstrating their importance in the food web even at relatively low abundance. Nevertheless, the EAA content of the trout juveniles did not differ between the treatments.

Previous laboratory results have shown that AA composition does not vary much among phytoplankton species (Ahlgren et al., 1992; Peltomaa et al., 2017). However, other studies have reported that the contribution of methionine, histidine, and tyrosine varied between phytoplankton taxa in a eutrophic reservoir (Kolmakova, Gladyshev, & Kalacheva, 2007). Phytoplankton AA composition Siberian reservoirs appear to be relatively unchanged throughout the open water season, except during the blooms of cyanobacteria that contain high amount of both EAA and NEAA (Ahlgren et al., 1992; Kalachova, Kolmakova, Gladyshev, Kravchuk, & Ivanova, 2004). Cyanobacteria may not be available to consumers as they frequently form filamentous aggregations, which are too large sized for filtering by herbivorous cladocerans. Therefore, it seems that the greatest challenges in EAA availability in pelagic food webs in eutrophic lakes are related to the contribution of the edible food particles for zooplankton and the following transfer of EAAs from phytoplankton–zooplankton trophic levels to fish. As bacteria contains equal EAAs to phytoplankton while tPOM is distinctly lacking in overall abundance of EAA, the EAA limitation of zooplankton in dystrophic lakes likely happens when zooplankton are forced to feed on terrestrial organic matter. These low nutritional quality periods can lead to decreased growth and reproduction of zooplankton due to the lack of both EAA and ω‐3 PUFA (Kleppel, Burkart, & Houchin, 1998; Peltomaa et al., 2017).

### 4.3 Accumulation and conversion of essential fatty acids (H2)

Our biochemical analysis showed little variation in the lipid and carbohydrate content of the basal foods and *Daphnia*, with their content exceeding the dietary requirements of juvenile rainbow trout. Furthermore, ALA and ω‐6 FA content in *Daphnia* exceeded or were close to the dietary requirements in all treatments. Both scenarios resulted in lower ALA and SDA content in the basal foods than eutrophication, and lower accumulation of ALA and SDA in the next two trophic levels. Thus, the second hypothesis that browning and eutrophication will reduce the accumulation of ω‐3 PUFAs was supported: browning decreased the abundance and transfer of EPA and DHA from basal foods via zooplankton to fish more than eutrophication. However, the EPA and DHA content were below the nutritional requirements for juvenile rainbow trout in all *Daphnia* treatments. Previous field studies on mesotrophic and eutrophic lakes have found that EPA deficiency limits *Daphnia* somatic growth and reproduction (Müller‐Navarra, 1995; Müller‐Navarra, Brett, Liston, & Goldman, 2000). This is mainly result of cyanobacteria replacing EPA and DHA synthesizing taxa in eutrophic lakes (Ravet, Persson, & Brett, 2012; Taipale, et al. 2016). Such changes in production of essential fatty acids could be potentially compensated by consumers by conversion of EPA and DHA from ALA.

Juvenile fish fed by artificially enriched fish feed were able to double their DHA content in 21 days, whereas in all *Daphnia* treatments fish lipid content remained at initial level. Contrary to our initial expectation, fish were unable to desaturate and elongate ALA into DHA in significant amounts. Nonetheless, through our ^13^CLE technique, some conversion was detected at trace levels in the highest ALA treatments but not in the low ALA treatment.

### 4.4 Growth and survival of fish juveniles (H3)

Previous studies have shown that the decrease in the nutritional quality of basal food sources (seston) due to the increase in tPOM, bacteria, or poor quality phytoplankton has negative effects on survival, growth, and reproduction of zooplankton (Brett, Kainz, et al. 2009; von Elert, Martin‐Creuzburg, & Le Coz, 2003; Taipale et al., 2014). In our three trophic level experiment, we studied whether browning and eutrophication also lowers survival and growth of fish juveniles. In accordance with our hypothesis, the decrease in nutritional quality of basal foods had cascading negative effects on juvenile fish as well. Furthermore, survival and growth of fish decreased under both browning scenarios more than under the eutrophication or hypereutrophication scenarios. Previous experiments have shown that a 1:1 mixing ratio of green algae and tPOM (as in our browning tPOM treatment) can support somatic growth of *Daphnia* (Taipale, et al. 2016). However, juvenile fish were unable to grow or even survive with *Daphnia* fed on this mixture, highlighting the poor nutritional value of tPOM for upper trophic levels. Our results also do not support the idea that tPOM can enhance the somatic growth of juvenile fish, but rather corroborates similar results obtained with a different fish species (*Esox lucius*; Sauvanet et al., 2013). This work compared various fatty acids in pike across multiple basal resources (seston, filamentous algae, epiphytes, macrophytes, and terrestrial tree litter) and found no evidence of terrestrial contribution (Sauvanet et al., 2013).

The growth of juvenile rainbow trout has been found to be highly variable between lakes (0.4%–1.6% per day) and correlate positively with DHA‐rich calanoid zooplankton (Knudson, 2011). Fish growth under our eutrophication scenarios were similar to the upper values found in nature (~1.6% per day) (Knudson, 2011). Nonetheless, they were still far behind the fish feed treatments that is optimized for fish growth, which suggests some effect of nutrient limitation on fish growth across all of our natural basal resource treatments. It must also be recognized that experimental and in‐nature measurements of fish growth are not directly comparable as experimental conditions never mimic nature exactly and are usually fixed at optimal temperature with excess food rations (as is the case here). The rainbow trout strains in our study have also been under breeding selection targeting for fast growth (Martens et al., 2014). Nonetheless, our results do indicate that fish development requires high‐quality phytoplankton (e.g., dinoflagellates, cryptophytes, chrysophytes, diatoms) providing nutritious feed for zooplankton and later satisfy the high nutritional demand of EAA and DHA by juvenile fish.

Difference in survival and growth between eutrophication and browning scenarios can be explained by particular importance of DHA for neural tissues especially during early stages of fish development (Mourente, Tocher, & Sargent, 1991). During this stage, DHA deficiency can cause an inadequate brain function and vision (Sargent, Bell, & Tocher, 1993), thus the juveniles obviously attempted to maintain high DHA content in their neural cells. The indication of trace amounts of ALA to DHA conversion and positive growth in fish under the high ALA treatments suggests that small amounts of conversion of ALA into DHA were crucial for keeping juveniles alive and supporting albeit slow growth. In contrast, under the low ALA treatment, there was no ALA to DHA conversion and associated with growth stagnation and reduced survival rate. According to previous studies, freshwater fish juveniles are considered to be able to grow with high ALA content due to their ability synthesize DHA (Sargent & Tacon, 1999). Our experiments showed that 4‐week‐old juvenile fishes cannot convert ALA into EPA and DHA sufficiently to meet their physiological demands (Wirth et al., 1997). Similarly, herbivorous zooplankton, both cladocera and calanoida, usually try to maintain high EPA and DHA content when dietary quality decreases (Koussoroplis, Nussbaumer, Arts, Guschina, & Kainz, 2014; Taipale et al., 2011). Altogether, our results showed that intake of DHA and its production at lower trophic levels is essential for juvenile rainbow trout.

### 4.5 Wider implications for food web quality

The Match–Mismatch hypothesis states that the recruitment of fish is governed by a match between the hatching of fish and the availability of plankton prey boom (Cushing, 1969). In nature, juvenile fish are size selective in the feeding, starting on small zooplankton including rotifers, copepod nauplii, and cladocerans (Perga et al., 2009; Turner, 2004). The EPA and DHA content of these zooplankton taxa varies greatly during the season mainly depending on succession among basal resources (i.e., phytoplankton and bacteria) and their own metabolic processes (Gladyshev, Sushchik, Dubovskaya, Makhutova, & Kalachova, 2006; Ravet, Brett, & Arhonditsis, 2010; Taipale et al., 2009). This research has demonstrated that phytoplankton nutritional quality with subsequent cascading effects on cladoceran nutritional quality and ultimately juvenile fish growth and survival. This suggests that bottom‐up processes which supply key biomolecules to consumers contribute to recruitment beyond simply the quantity of prey.

There is indeed some recent evidence that browning of lakes induced shifts toward increased use of terrestrial organic matter by primary consumers that reduced biomass and production of Eurasian perch (*Perca fluviatilis*; Karlsson et al., 2015). Our results show that juvenile fish growth can be completely ceased in the absence of one biomolecule, DHA, even though other nutritional requirements are met. This fits with previous results from aquaculture, where high DHA content enhances development and growth of juvenile fish (Harel et al., 2002; Trushenski, Schwarz, Bergman, Rombenso, & Delbos, 2012). Our experimental results from the three trophic levels together with previous field data (Taipale, et al. 2016) demonstrate that high ALA content cannot be DHA in sufficient quantity to maintain fish growth. To conclude, the eutrophication and browning induced shifts in phytoplankton community composition reduce production of DHA, resulting in lower transfer rates to zooplankton and fish suggesting the bottom‐up regulation of food web quality. From the previous observations, we anticipate that these results to be transferable real food webs in natural lakes, although further whole‐ecosystem experiments are necessary to order to be conclusive.

## CONFLICT OF INTEREST

None declared.

## AUTHOR CONTRIBUTIONS

SJT conceived the initial idea, conducted the experiments, and wrote the first draft of manuscript. EP and KKK contributed to idea development and participated in sample analysis and manuscript preparation. GWH provided compound‐specific isotope analysis and helped with finalize revisions of the manuscript.
